# Non-Invasive Detection of a Small Number of Bioluminescent Cancer Cells *In Vivo*

**DOI:** 10.1371/journal.pone.0009364

**Published:** 2010-02-23

**Authors:** Jae-Beom Kim, Konnie Urban, Edward Cochran, Steve Lee, Angel Ang, Bradley Rice, Adam Bata, Kenneth Campbell, Richard Coffee, Alex Gorodinsky, Zhan Lu, He Zhou, Takashi Kei Kishimoto, Peter Lassota

**Affiliations:** 1 Caliper Life Sciences Inc., Alameda, California, United States of America; 2 Caliper Life Sciences Inc., Cranbury, New Jersey, United States of America; 3 Momenta Pharmaceuticals Inc., Cambridge, Massachusetts, United States of America; Garvan Institute of Medical Research, Australia

## Abstract

Early detection of tumors can significantly improve the outcome of tumor treatment. One of the most frequently asked questions in cancer imaging is how many cells can be detected non-invasively in a live animal. Although many factors limit such detection, increasing the light emission from cells is one of the most effective ways of overcoming these limitations. Here, we describe development and utilization of a lentiviral vector containing enhanced firefly luciferase (*luc2*) gene. The resulting single cell clones of the mouse mammary gland tumor (4T1-luc2) showed stable light emission in the range of 10,000 photons/sec/cell. In some cases individual 4T1-luc2 cells inserted under the skin of a *nu/nu* mouse could be detected non-invasively using a cooled CCD camera in some cases. In addition, we showed that only few cells are needed to develop tumors in these mice and tumor progression can be monitored right after the cells are implanted. Significantly higher luciferase activity in these cells allowed us to detect micrometastases in both, syngeneic Balb/c and *nu/nu* mice.

## Introduction

Detection of tumors at early stages is critical for effective tumor treatment and for studying tumorigenesis[1], [2], [3]. Traditionally, tumor growth was assessed by using mechanical or electronic calipers to take physical measurements of subcutaneous human tumors growing in immunocompromised mice[4]. This method is suitable, however, only for palpable tumors growing under the skin of the animals. Deeper tumor masses, such as osteosarcomas encapsulated by the bone, are not amenable to direct physical measurements. Even in subcutaneous models, tumor burdens may not be accurately quantified using physical measurements because edema and necrotic centers will contribute to the increase in tumor size[5]. Orthotopic solid tumor models circumvent these obstacles and allow fairly accurate assessment of tumor burdens by weighing the excised, “cleaned” tumors after the animals are sacrificed. Classical orthotopic models are impractical for evaluation of compounds' efficacy since they require large numbers of animals to be sacrificed at each time point. Similarly, identification of tumors and quantification of tumor burden in models of metastasis demand exhaustive and tedious histological analyses[6], [7], [8].

Non-invasive whole body bioluminescence imaging (BLI) allows repeated, real-time *in vivo* monitoring of tumor growth in experimental animals, regardless of tumor locations. In contrast to fluorescence, BLI exhibits minimal background signals from the animal tissues[9]. Therefore, BLI can detect relatively weak signals with high signal to background ratio. Due to its versatility, BLI has been adopted to study preclinical efficacy of drug candidates[10], [11], [12], [13] as well as various aspects of mammalian biology via reporter assays[14], [15].

Recently, firefly (*Photinus pyralis*) luciferase was re-engineered to further optimize its expression in mammalian cells. Compared to previous generations of luciferase, the new version (*luc2*) delivers more than a four-fold increase in light emission which was achieved by codon optimization and removal of potential transcription factor binding sites[16]. We postulated that individual cancer cells could be detected *in vivo* by harnessing the increased bioluminescence of *luc2*. To that end, we engineered a lentiviral vector where *luc2* expression is driven via the human ubiquitin C promoter[17]. The construct was then stably transfected into the 4T1 mouse mammary tumor cell line[18], [19]. Several stable, single-cell clones (4T1-luc2) were subsequently isolated with light emission in the range of 10,000 photons/sec/cell. Here, we report that, at least in some cases, detection of a single cancer cell *in vivo* using one of these luc-2 labeled clones was achieved. We also show development of tumors from only few cells implanted into *nu/nu* mice and detection of metastases in syngeneic Balb/c mice. To our best knowledge, this is the first report of detection of a single bioluminescent cell *in vivo* using a non-invasive imaging method. Such bright cells can be effectively used to monitor efficacy of drug candidates in models of metastasis and orthotopic tumor models, to track metastatic migration of cancer cells, and can be also utilized to accurately ascertain whether any residual disease remains following the treatment.

## Results

### Generation of a Lentiviral Vector System and Stable 4T1-luc2 Cell Lines

A lentiviral vector containing the firefly *luc2* gene conjugated to a human ubiquitin C promoter was constructed to generate stable bioluminescent cancer cell lines[16], [17], [18]. Mouse mammary tumor 4T1 cells were then transfected with the lentiviral vector and stable clones were selected using puromycin (4T1-luc2). Eight clones were chosen for further analyses and their luciferase activities were monitored for four weeks without selection marker. Although there were variations among the clones, the majority of them clones emitted more than 3,000 photons/sec/cell of light. Considering that most cell lines labeled with previous generations of luciferase emit less than 250 photons/sec/cell, our luc2 clones exhibited considerably higher level of light emission[20]. Surprisingly, one clone (C26) initially emitted as much as 52,000 photons/sec/cell but its light emission decreased to 6,400 photons/sec/cell after four weeks (Figure S1A). To confirm that no alteration of cellular physiology occurred during the labeling/cloning process, we compared the clones to the original parental 4T1 cells on several different levels. First, we examined growth patterns. From the eight initially selected clones, we chose two lines (C27 and C38) and compared their growth patterns to the parental 4T1 cells. Both lines had similar doubling times to the parental cells (12.6 hour doubling time for both clones, versus 12.0 hours for the original 4T1 cells).

We also examined other critical parameters of cellular physiology, including effects of ATP consumption. Since luciferase uses one molecule of ATP to produce each photon of light, high levels of light emission could be detrimental to the cell's metabolism due to the depletion of the its ATP pool. To test whether the high light production affects cell physiology, we observed cell growth for four days in the presence of high concentrations of the luciferase substrate, D-luciferin (150 and 300 µg/ml/day). The results demonstrated that, in the presence of D-luciferin, 4T1-luc2 clones showed similar growth patterns to those of cells cultured without D-luciferin and to the parental 4T1 cells (Figure S1B,C,D). This suggests that 4T1-luc2 cells can endure consumption of the ATP required for the high light emission without a significant effect on the cell's ATP pool.

**Figure 1:**
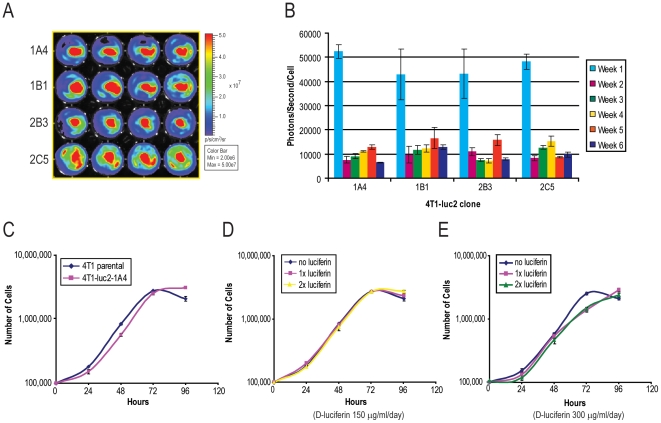
Generation of 4T1-luc2-1A4 cells and their growth patterns in the presence or absence of D-luciferin. (A) Generation of 4T1-luc2-1A4 cells. Mouse mammary tumor 4T1 cells were transfected with a lentiviral vector containing enhanced luciferase 2 under control of the human ubiquitin C promoter[16], [18]. Puromycin-resistant clones were isolated and their luciferase expressions were screened by bioluminescence. Two rounds of cloning generated 4 single cell clones of 4T1-luc2. Luciferase activities were measured using an IVIS Spectrum (Binning: med, f stop: 1, exposure time: 1 sec). A typical bioluminescence image for testing stability of luciferase expression is shown. Total flux (photons/sec) was quantified using Living Image software 3.0. Clone 4T1-luc2-1A4 was selected and used for further studies. (B) Stability of luciferase activity of four 4T1-luc2 clones. Cells were grown for 6 weeks in regular media without puromycin and their light emission was monitored weekly. All clones showed more than 7,000 photons/sec/cell of light emission throughout the test period. (C) Growth curves of the 4T1-luc2-1A4 clone and parental 4T1 cells. Cells were grown for 4 days in regular growth medium without puromycin. Total numbers of cells over time were plotted in a logarithmic scale. Both cell lines showed similar growth patterns and doubling times. (D,E) Growth of the 4T1-luc2-1A4 clone and parental 4T1 cells in the presence of D-luciferin. Cells were fed with D-luciferin once a day (150 µg/ml/day, D) or twice a day (300 µg/ml/day, E), respectively, harvested at each time point and counted. Presence of the excess of D-luciferin did not affect the overall growth patterns of the 4T1-luc2 cells.

Since clone C26 showed decreasing luciferase activity over time, we attempted the second round of limited dilution single cell cloning from the original mixed population. Four bright clones were selected and their luciferase activities (light emission) were monitored for six weeks without selection pressure (Figure 1A,B). All clones initially produced more than 40,000 photons/sec/cell; then the light emission declined to 10,000 photons/sec/cell, and stabilized at that level. It remained stable for four weeks in the absence of puromycin (Figure 1B). From these clones, the 1A4 clone (4T1-luc2-1A4) was selected for further studies. The growth pattern of the 4T1-luc2-1A4 was comparable to that of the parental 4T1 cells in the presence, or absence of D-luciferin (Figure 1C,D,E). To address the cause of the initial decrease of light emission in 4T1-luc2-1A4 clone, we performed limited dilution culture in 96-well plates. When cells grew to about 25% confluency, we examined luciferase expression by bioluminescent imaging. Every well containing cells showed luciferase activity. These results indicate that the initial decrease of luciferase activity was not due to the loss of luciferase expression in some cells of the clone (Figure S2).

### Non-Invasive Detection of Small Numbers of Cells in *nu/nu* Mice

**Figure 2:**
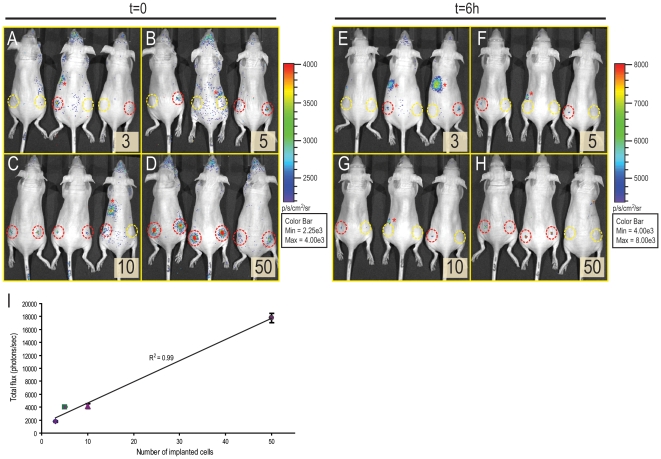
Detection of small numbers of 4T1-luc2-1A4 cells *in vivo*. (A-D) Defined numbers of cells were implanted subcutaneously in dorsal flank regions of female nu/nu mice[1]. Each mouse received two implantations. Insets indicate the number of cells implanted. D-luciferin was injected into mice immediately after the implantation and bioluminescent images were taken (t = 0) using an IVIS Spectrum (FOV; A, binning; small, f stop; 1, exposure time; 5 min). (E-H) After 6 hours of implantation, all mice were re-imaged using the same settings of IVIS Spectrum (t = 6h). Red circles represent the implantation sites that had bioluminescent signals higher than autoluminescence. Yellow circles indicate the implantation sites that did not generate any meaningful signals possibly due to immediate cell death. (I) Correlation between the number of implanted cells and the total flux from the implantation sites. Bioluminescent signals were quantitated using Living Image software 3.0 and plotted against the numbers of cells. The measured intensity of bioluminescence was directly proportional to the number of implanted cells. Asterisks (*) indicate tissue autoluminescence.

Because 4T1-luc2 cells showed extremely high light emission, we next attempted to detect small numbers of these cells *in vivo*. Initially, the 4T1-luc2-1A4 cells were prepared using a serial dilution method and were implanted into both flanks of the female nu/nu mice (Figure 2). Different numbers of cells were implanted at each implantation sites. Six implantations were performed for each number of cells (3, 5, 10, and 50 cells). Bioluminescence images were taken immediately after the implantations. Using a highly sensitive cooled CCD camera, we were able to detect as few as 3 cells in this experiment (Figure 2A-D, red dotted circles). In some instances, however, we did not detect any meaningful signals from the sites of implantation (Fig 2A-D, yellow dotted circles). This could be attributed to cell death immediately after implantation or to inherent variability of the serial dilution method when the intended number of cells is very small (Fig 2A-D, yellow circles). Separately, we took PC3M-luc-C6 which was one of the brightest cell lines (∼250 photons/sec/cell) generated by us thus far (using multiple rounds of transfections by pGL3) and compared it with 4T1-luc2-1A4 cells, by implanting both into SCID-bg mice (Figure S3). The results illustrate that the bioluminescent signals from 10^2^ 4T1-luc2-1A4 cells in furry mice was easily detectable, while no signal was detected from the same number of PC3M-luc-C6 cells.

Six hours after the implantations, we re-imaged the same set of animals (Fig 2E-H). As expected, based on the hostile post-implantation environment, 4 sites of the 10- and 50 cell implantation sites lost their initial bioluminescent signals (Fig 2G,H). Surprisingly, however, we were able to detect signals from 3- and 5 cell implantation sites (Figure 2E,F). Our analyses of the images taken immediately after the implantation (t = 0) indicate that total flux from the implantation sites was directly proportional to the number of cells implanted (Fig 2I). This is consistent with the other data (not shown) demonstrating linear relationship between the light emission and the number of cells plated *in vitro*. Based on these results, we demonstrated that bioluminescence measurement is a plausible method to accomplish non-invasive monitoring of the early tumor growth *in vivo*.

### Detection of a Single Bioluminescent 4T1-luc2 Cell *In Vivo*

**Figure 3:**
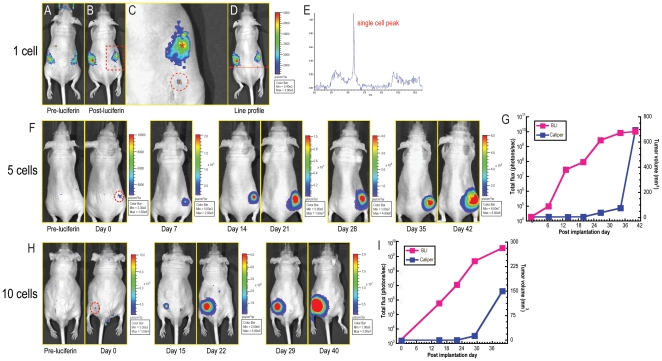
Detection of a single 4T1-luc2-1A4 cell *in vivo*. (A-C) Bioluminescent signal of a single 4T1-luc2-1A4 cell *in vivo*. A single cell was implanted into the back of a *nu/nu* mouse. D-luciferin was injected into the mouse intraperitoneally and bioluminescent images were taken using an IVIS Spectrum (FOV; C, binning; small, f stop; 1, exposure time; 5 min). Images for pre- and post-luciferin injection were shown in panels (A) and (B), respectively. Magnified image of the dotted area from panel (B) is shown on panel (C). The dotted circle represents the single cell signal. The asterisk (*) indicates the background signal from the gut. (D,E) Line profiling analysis of single cell signal. Light emission was plotted along the line shown on panel (D). Peak signal in the panel (E) represents the light emission from a single 4T1-luc2-1A4 cell. (F) Tumor development from five 4T1-luc2-1A4 cells. Cells were implanted subcutaneously (using a micropipette) into the dorsal flank region of a *nu/nu* mouse. Bioluminescent images were first taken before the D-luciferin injection (Pre-luciferin). The animal was then imaged on day 0 through day 42. (G) Monitoring of tumor growth from 5 cells of 4T1-luc2-1A4. Bioluminescent signals were quantified using Living Image software and plotted against physical tumor volume measurements by a caliper. Tumor was not palpable till day 27 post-implantation while bioluminescent signals were detected from the day 0. Note that total flux was plotted in a logarithmic scale. (H) Tumor development from 10 cells of 4T1-luc2-1A4. Cells were implanted subcutaneously (using a micropipette) into the back of a *nu/nu* mouse. The background signal is shown in pre-D-luciferin injection image (Pre-luciferin). The tumor growth was monitored for 40 days using an IVIS Spectrum and a caliper. (I) Monitoring of tumor growth from 10 cells of 4T1-luc2-1A4. Tumor volumes were measured using a caliper and plotted against bioluminescent signals which were quantified using Living Image software. Tumor was not palpable till day 29 after implantation. On the contrary, bioluminescent signals were distinct from the day 0 of implantation.

After confirming non-invasive detection of three cells *in vivo*, we challenged ourselves to detect a single 4T1-luc2-1A4 cell after subcutaneous implantation. To eliminate the experimental error and to add accuracy to the determination of the number of implanted cells, we used a micropipettor to implant a single 4T1-luc-2-1A4 cell. First, the 4T1-luc2-1A4 cells were trypsinized and plated on a cell culture dish. Next, individual cells were picked up and implanted using a micropipettor into subcutaneous slots made in the flank regions of mice. Mice were divided into two groups: four mice were implanted with single cells, and four other mice were implanted with 10 cells each (Figure S4C,D). Animals were then subjected to bioluminescence imaging immediately after the implantation. In some cases, we were able to detect a single 4T1-luc2-1A4 cell (Figure 3A-C and Figure S4A,B). On each of the three independent, sequential images of the same single cell we registered a total flux ranging from 460 to 528 photons/sec. Line profiling analyses of the registered flux from a single cell revealed a signal to background ratio of 6 to 1, with the signal clearly originating from the implantation site (Figure 3D,E). Therefore, we concluded that the bioluminescent signal indeed originated from a single 4T1-luc2-1A4 cell. The lack of signal from the other three sites in each group could be attributed to rapid cell death.

### Tumor Development from Small Populations of 4T1-luc2 Cells

After detecting a single cell *in vivo*, the mice implanted with 1–50 cells were monitored for extended period of time to detect possible tumor growth. We hypothesized that implantation of larger numbers of cells would circumvent the problem that hostile post-implantation environments present to smaller numbers of cells. Given that routine tumor implantation procedures utilize 0.5 to 10 millions of cells in subcutaneous tumor models, we did not expect tumors to arise from such small numbers of cells. To our surprise, two mice that were implanted with 5 and 10 cells developed solid tumors. We continued to image these mice, and once the tumors became palpable, we also physically measured their dimensions using standard calipers. The tumors arising from 5-cell and 10-cell implantations could not be detected with calipers before day 27 and day 29, respectively (Figure 3G,I). However, non-invasive visible light imaging, allowed us to detect and quantify tumor burdens continuously from the time of implantation (Figure 3F,H). These data clearly show that tumor growth can be monitored using non-invasive bioluminescence imaging as soon as cells are implanted in an animal, even when as few as five cells are implanted.

### Metastases of 4T1-luc2-1A4 Cells in Syngeneic Balb/c Mice from the Orthotopic Implantation into the Mammary Fat Pad

**Figure 4:**
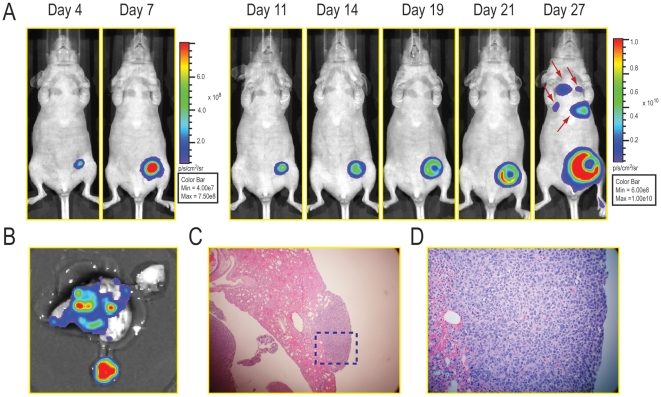
Non invasive detection of micrometastases and histological analysis. (A) Female nu/nu mice were inoculated with 5×10^5^ 4T1-luc2-1A4 cells orthotopically into the abdominal mammary fat pads[1]. Bioluminescent images were taken longitudinally. At post-implantation day 27, micrometastases were detected in lungs (arrows) (B) The lungs were isolated at post-implantation day 27 and *ex vivo* image was taken. (C,D) Lung tissues were fixed in formalin and embedded in paraffin. H&E staining was performed and analyzed. Panel D represents the dotted area in panel C.

To test metastatic properties of the 4T1-luc2-1A4 cells, we orthotopically implanted these cells into mammary fat pads of female nu/nu mice (5×10^5^ cells per mouse, n = 9). The primary tumors grew rapidly and developed metastatic lesions that could be detected using bioluminescence imaging by day 27 (Figure 4). To confirm metastasis of tumor cells into lungs, we isolated lung tissues on day 27 post-implantation and took *ex vivo* images (Figure 4B). In addition, we performed histological analyses on formalin-preserved, paraffin sectioned tissues. The results showed that pleura and subpleural regions of the lungs were infiltrated with sheets of poorly differentiated neoplastic cells demonstrating that bioluminescent imaging can effectively detect micrometastases in a mouse (Figure 4C,D). However, it is difficult to speculate exactly how many cells resided in the metastasized tumor masses based on the light emission registered *in vivo* since the emitted light is attenuated and scattered depending on path it takes through the tissues, and the exact location of these tumors has not been elucidated.

**Figure 5:**
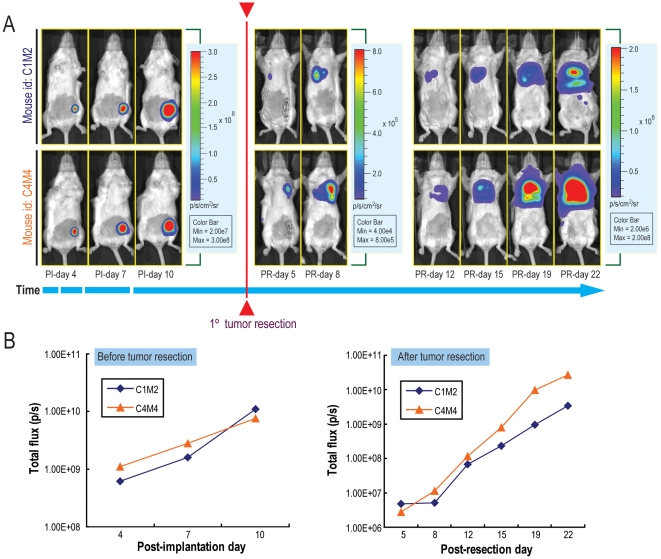
4T1-luc2-1A4 tumor metastases in syngeneic Balb/c mice. (A) Metastases of 4T1-luc2-1A4 tumors in Balb/c mice. 5.0×10^4^ cells were orthotopically implanted into mammary fat pads of the mice (n = 16)[1]. Ventral Images were taken at three time points post-implantation (PI-days: 4, 7, 8). Primary tumors were resected at PI-day 10 and images were taken again at various post-resection time points (PR-days: 5, 8, 12, 15, 19, 22). Two representative mice (C1M2 and C4M4) are shown. The apparent decrease of the bioluminescence signals at PR-day 12 was due to the adjustment of the color bar scale (see panel B). Primary tumor resection time point is indicated by the red line separating the pre-and pot-resection images. (B) Plots of bioluminescence signals vs. time for mice C1M2 and C4M4. Whole body bioluminescence signals of mice C1M2 (blue lines) and C4M4 (orange lines) were quantitated and plotted in a logarithmic scale. Quantitations of bioluminescence signals before and after resection of the primary tumors are shown.

Next, we confirmed the detection of metastases by bioluminescence via physical dissection. We created a second group of Balb/c mice, into whose mammary fat pads we implanted 5.0×10^4^ 4T1-luc2-1A4 cells (n = 16). Primary tumors were then resected at post-implantation day 10 to stop the growth of the tumors in the fat pads, and bioluminescent images were taken at various post-resection (PR) time points (PR-day 5, 8, 12, 15, 19, 22). Our data showed that tumors metastasized into the secondary sites in the body and continued to grow there (Figure 5A). Tumor growth was monitored longitudinally by quantitating bioluminescence signals from the whole body (Figure 5B). The results demonstrated continuous increase of the light emission before and after resection of the primary tumors, confirming that monitoring bioluminescence signals is an ideal way to track tumor metastases.

## Discussion

Xenografting of luciferase-labeled cancer cells is widely accepted in models of metastasis and in orthotopic models. As discussed above, bioluminescent imaging of luciferase-labeled cancer cells has the added advantage over traditional methods of assessing tumor burden in that it allows non-invasive detection and quantitation of tumors in live animals as means of assessing drug efficacy[21], [22], [23]. Despite the fact that tissues normally contribute little background in bioluminescence imaging, increasing the light emission from the cells of interest is always desirable since it improves the sensitivity of detecting tumor cells. Increased sensitivity allows smaller numbers of tumor cells present in early stages of tumor progression to be detected. This conceivably has significant clinical relevance given that early detection, when combined with early treatment, has been correlated with better prognoses. The tools described here allow one to compare the effectiveness of a given pharmacological intervention on early stage primary tumors, late stage primary tumors, and metastases.

Herein we report development of a bright 4T1-luc2 cell line using enhanced luciferase (*luc2*) and lentiviral technology. In our attempts to detect small numbers of cells, we initially used a serial dilution method and could detect down to 3 cells *in vivo*. Encouraged by these results, we challenged ourselves to detect a single 4T1-luc2-1A4 cell after subcutaneous implantation via microinjection. Because the signal from a cell was located in proximity to the gut, which exhibits an intrinsic, albeit variable auto-bioluminescence, our images of single cells contain both, signals from the cell, as well as background from the gut (Figure 3B). However, as shown on the images of 5- and 10- cells, when the numbers of cells increased, the signals from the cells quickly surpassed the background signals from the gut. During the course of preparation of this manuscript, Rabinovich *et al*. showed the detection of three engineered murine T lymphocytes *in vivo* in a subcutaneous transplantation[24]. While application of the elegant system described by Rabinovich *et al*. was developed to achieve efficient transduction for a specific cell subtype, we have engineered a simple, universal vector to transduce various types of proliferating and non-proliferating cells. Moreover, to the best of our knowledge, our report is the first one that shows detection of a single bioluminescent cancer cell *in vivo*.

Because high level of luciferase expression increases the sensitivity of cell detection in live animals, this technology can be directly applied to primary cell and stem cell detection *in vivo*, including cancer stem cells[25], [26], [27]. The lentivirus technology can conceivably prove useful for labeling stem cells since it can significantly minimize handling and culturing of these cells. Our results showed that as few as 5 and 10 cells can grow and form tumors in the animals. These tumors can be monitored using bioluminescence imaging from the day of implantation. Previously, 4T1 cells were transformed with luciferase and their tumor metastases were visualized using optical imaging[28]. While this study demonstrated non-invasive monitoring of tumor metastases, the present study enables earlier detection of tumor metastases, and allows following the tumor formation process right from the cell implantation (Day 0 vs. 6 weeks). Furthermore, our results suggest that labeling and tracking cancers growing from a single cancer stem cell is feasible. In addition, this technology could also be applied more generally to follow the fate of a single stem cell implanted into an animal. Furthermore, the process of drug screening for either small molecules or biologics which target cancer stem cells can be significantly expedited since bioluminescence allows following growth of tumors *in vivo* weeks before they become palpable.

Brightly luminescent cells also provide a better means of detection of micrometastases in an animal, thus making models of metastasis more accurate and allowing predictive models to be used for evaluation of drug candidates that aim to combat metastasis. When 4T1-luc2-1A4 cells were implanted into syngeneic Balb/c mice, we were able to detect multiple micrometastases in secondary sites. When primary tumors were surgically removed at 27 days after the implantation, we could follow the growth of small, metastatic tumors in the absence of the dominating signal from the primary tumor. Brighter cells can reduce the time and effort required to identify tumor masses in an animal and can also be valuable in studying tumor microenvironments, particularly when combined with other non-invasive fluorescence-based readouts.

To date, we have labeled more than a dozen human and murine tumor cell lines using the described lentiviral system (data not shown). All labeled cell lines showed significantly higher luciferase expression compared to the cell lines labeled with previous generation of luciferase and conventional transfection methods (data not shown). Another advantage of these newly engineered cell lines is that no antibiotic selection is required to maintain stable luciferase expression. In contrast to the popular viral promoters such as SV40 or CMV, human ubiquitin C promoter is more resistant to gene silencing in mammalian cells[29]. We have used our technology to successfully label adherent, as well as suspension cell lines, and we believe that it constitutes a universal tool for efficient introduction of luciferase into almost any cell. Since lentiviral vectors can introduce genes of interest into dividing as well as non-dividing cells, our technology can be easily applied to label not only stem cells, but practically any cells derived from patients, which then can be used for research, or for diagnostic purposes.

## Materials and Methods

### Generation of Lentivirus Vector

Enhanced luciferase 2 (*luc2*) cDNA was from pGL4.20 vector (Promega, WI)[16]. Luciferase 2 cDNA was excised with Hind III & Xba I and ligated into pUB6-V5-HisB vector (Invitrogen, CA). A fragment generated by Bgl II and BstB I digestion of pUB6-luc2 was ligated into a modified pLPCX vector (Clontech, CA). A lentiviral vector that carries human ubiquitin C promoter and luc2 cDNA was generated by inserting Bgl II & EcoR I fragment from above construct into Bcl I & EcoR I of the modified pLKO.1 vector (Sigma-Aldrich, MO)[18], [19].

### Cell Culture

Mouse mammary gland tumor cell line 4T1 was obtained from the ATCC (Manassas, VA). Cells were grown in high glucose RPMI 1640 medium (ATCC). PC-3M-luc-C6 cells were grown in minimal essential medium (ATCC) (For supplemental data)[30]. All media was supplemented with 10% fetal bovine serum (Hyclone, UT) without antibiotics. Growth curves were generated by seeding 50,000 cells in T25 flasks. At each time point, cells were trypsinized and counted using an automatic cell counter (Nexcelom, MA). Total numbers of cells were plotted in a logarithmic scale.

### Transfection and Stable Cell Line Generation

The lentiviral vector was transfected using a lipid based method into 4T1 cells. Transfected cells were selected using puromycin (2 µg/ml). Isolated clones were screened for their luciferase activities using an IVIS Spectrum (Caliper Life Sciences, MA). To isolate single cell clones, cells were subjected to limited dilution. Individual clones were screened for luciferase activity using an IVIS® Spectrum. Selected clones were maintained without puromycin for 4 weeks and their light emission was monitored every week.

### Mice and Tumor Cell Implantation

All procedures for animal care and tumor cell implantation followed the approved animal protocols and guidelines of the Institutional Animal Care and Use Committee at Caliper Life Sciences and Momenta Pharmaceuticals. Prior to implantation, all tumor cells tested negative for the presence of mycoplasma and mouse pathogens. The orthotopic implantation of 4T1-luc2-1A4 cells into mammary fat pads of *nu/nu* or Balb/c mice was performed while animals were under isoflurane anesthesia[1]. Subcutaneous implantations were done by injecting cells under the skin in the dorsal flank regions. Single, and ten cell implantation was performed using a customized glass capillary pipette with manual aspiration. After the implantation, pipettes were examined under the microscope to make sure that all cells were implanted. Four implantations were done for each, one and ten cell implantations. For the data shown on Figure 2, six implantations were performed, three mice per each of the two groups.

### *In Vitro* and *In Vivo* Bioluminescence Imaging

For *in vitro* luciferase assay, cells were plated on black walled 24-well plates at an initial concentration of 50,000 cells/well. Cells were grown overnight with regular growth medium. After 24 hours, the regular medium was replaced with the D-luciferin containing medium (150 µg/ml). Bioluminescence images were taken immediately after adding the substrate into the cells using an IVIS Spectrum. Light outputs were quantified using Living Image 3.0 (Caliper Life Sciences, Alameda, CA). Prior to the *in vivo* imaging, the mice were anesthetized with isoflurane. D-luciferin solution was then injected intraperitoneally (150 mg/kg). The mice were imaged using an IVIS Spectrum. Bioluminescent signals were quantified using Living Image 3.0 (Caliper Life Sciences, Alameda, CA).

### Histological Analyses

Female nu/nu mice were inoculated with 5×10^5^ 4T1-luc2-1A4 cells orthotopically into the abdominal mammary fat pad (n = 9). At day 27 post-implantation, lung tissues were isolated and analyzed histopathologically. Tissues were fixed and embedded in paraffin. H&E staining was performed. Slides were examined by a certified pathologist.

## Supporting Information

Figure S1 (A) Generation of 4T1-luc2 cells. Mouse mammary tumor 4T1 cells were transfected with a lentiviral vector containing enhanced luciferase 2[18], [31]. Puromycin resistant clones were isolated and their luciferase expression was screened by bioluminescence. Initial cloning generated 8 clones of 4T1-luc2. Luciferase activity was measured using an IVIS Spectrum (Binning: med, f stop: 1, exposure time: 1 sec). Total flux (photons/sec) was quantified using Living Image software 3.0. Stability of luciferase activities of the 4T1-luc2 clones were monitored for 4 weeks and their light emission was measured weekly. All clones showed higher than 3,000 photons/sec/cell of the light emission throughout the test period. (B) Growth curves of the 4T1-luc2-C27 and the 4T1-luc2-C38 clones vs. parental 4T1 cells. The cells were grown for 4 days in a regular growth medium without puromycin. The total numbers of cells over time are plotted in a logarithmic scale. Both cell lines showed similar growth patterns and doubling times. (C,D) Growth of the 4T1-luc2-C26 and the 4T1-luc2-C36 clones vs. parental 4T1 cells in the presence of D-luciferin. The cells were fed with D-luciferin once a day (150 µg/ml/day, C) or twice a day (300 µg/ml/day, D), respectively. The cells were harvested at each time point and counted. Presence of excess of D-luciferin did not affect the overall growth patterns of the 4T1-luc2 cells.
(1.59 MB TIF)

Figure S2 (A) Limited dilution culture was performed with 4T1-luc2-1A4 cells in four 96-well plates. Cells were grown for 10 days and examined their luciferase expression by adding D-luciferin into the culture media. Bioluminescent images were taken immediately. Wells that did not show any luciferase activity did not contain live cells.
(5.36 MB TIF)

Figure S3 (A-D) The 4T1-luc2-C26 and the PC3M-luc-C6 cells were subcutaneously implanted into flank regions of SCID-bg mice. Equal numbers of cells for each cell line was implanted. Bioluminescence images were taken 20 hrs post-implantation using an IVIS Spectrum. Numbers of implanted cells are shown on the inserts. Imaging conditions (A,B; FOV: B, binning: small, f stop: 1, exposure time: 30 sec; C,D; FOV: B, binning: small, f stop: 1, exposure time: 5 min).
(2.72 MB TIF)

Figure S4 (A, B) Bioluminescent signal of a single 4T1-luc2-1A4 cell *in vivo*. Female *nu/nu* mouse was implanted with a single 4T1-luc2 cell subcutaneously in the dorsal region. Mouse was imaged prior to D-luciferin injection (A). Ten minutes after the D-luciferin injection, whole mouse image was taken (FOV; C, binning; small, f stop; 1, exposure time; 5 min) (B). Dotted circle indicates the signal from the implanted cell. (C) Whole mouse (*nu/nu*) image with implanted ten 4T1-luc2-1A4 cells. The exact number of cells was picked up by a glass capillary pipet and was injected into the back of the mouse subcutaneously, through a skin incision. (D) Magnified image of dotted area from panel C. Dotted circle indicates the signal from 10 cells. Asterisk (*) indicates the skin incision site.
(3.57 MB TIF)
