# Supplementary figures and images for: Non-Invasive Detection of a Small Number of Bioluminescent Cancer Cells In Vivo
Source: PLoS One. 2010 Feb 23;5(2):e9364. doi: 10.1371/journal.pone.0009364 (PMC2826408; doi:10.1371/journal.pone.0009364)

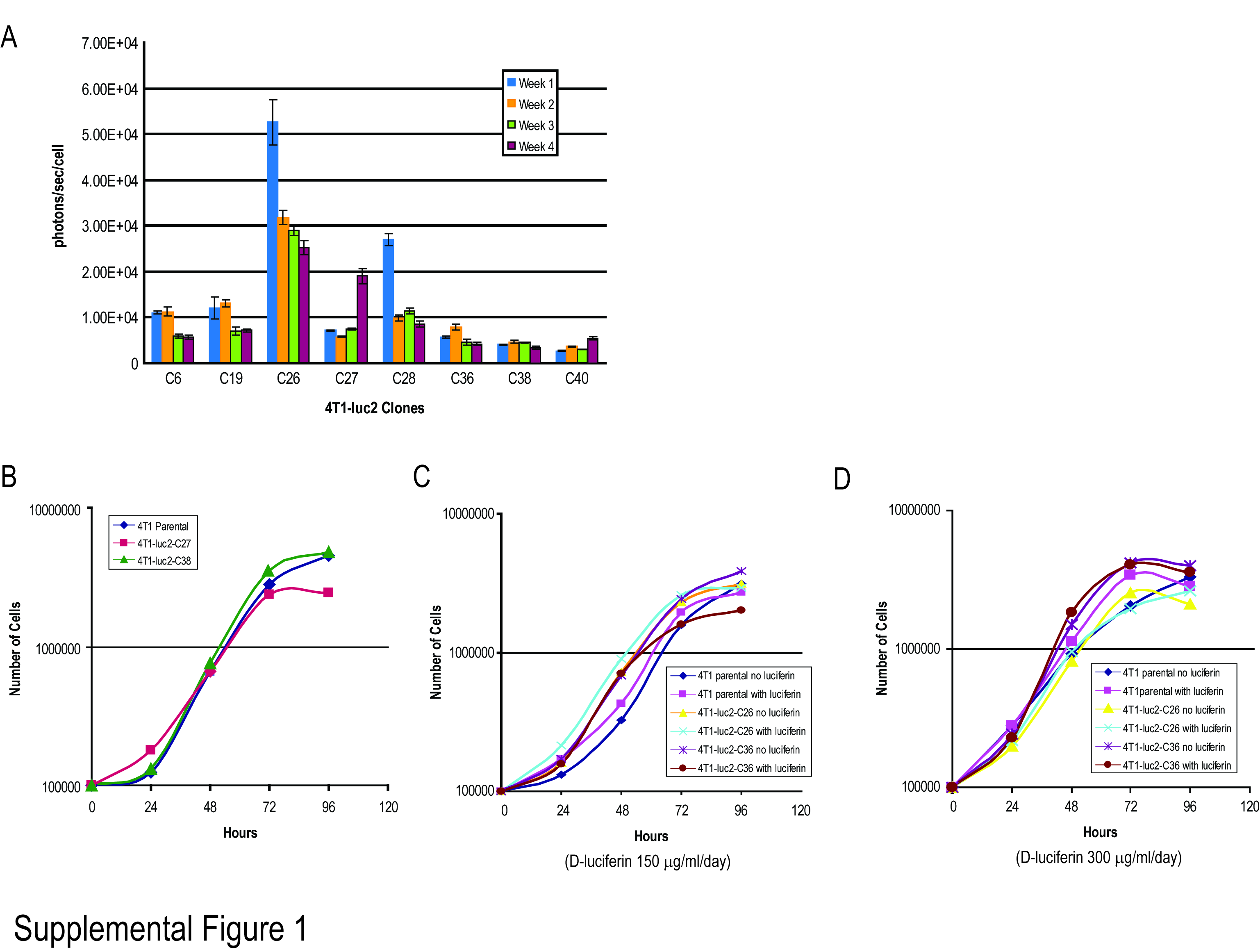

Supplement: Figure S1 — (A) Generation of 4T1-luc2 cells. Mouse mammary tumor 4T1 cells were transfected with a lentiviral vector containing enhanced luciferase 2[18], [31]. Puromycin resistant clones were isolated and their luciferase expression was screened by bioluminescence. Initial cloning generated 8 clones of 4T1-luc2. Luciferase activity was measured using an IVIS Spectrum (Binning: med, f stop: 1, exposure time: 1 sec). Total flux (photons/sec) was quantified using Living Image software 3.0. Stability of luciferase activities of the 4T1-luc2 clones were monitored for 4 weeks and their light emission was measured weekly. All clones showed higher than 3,000 photons/sec/cell of the light emission throughout the test period. (B) Growth curves of the 4T1-luc2-C27 and the 4T1-luc2-C38 clones vs. parental 4T1 cells. The cells were grown for 4 days in a regular growth medium without puromycin. The total numbers of cells over time are plotted in a logarithmic scale. Both cell lines showed similar growth patterns and doubling times. (C,D) Growth of the 4T1-luc2-C26 and the 4T1-luc2-C36 clones vs. parental 4T1 cells in the presence of D-luciferin. The cells were fed with D-luciferin once a day (150 µg/ml/day, C) or twice a day (300 µg/ml/day, D), respectively. The cells were harvested at each time point and counted. Presence of excess of D-luciferin did not affect the overall growth patterns of the 4T1-luc2 cells. (1.59 MB TIF) [file pone.0009364.s001.tif]

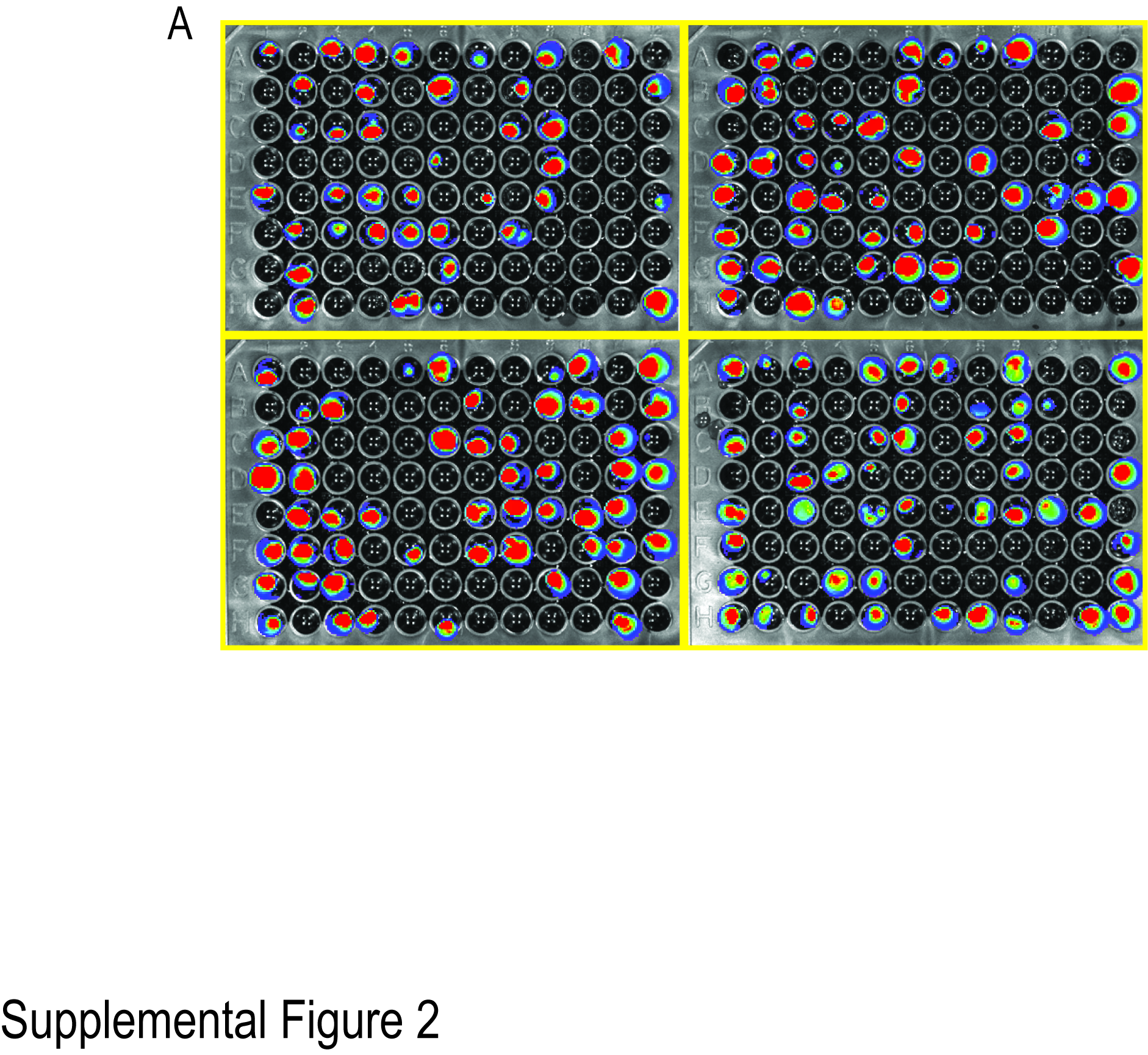

Supplement: Figure S2 — (A) Limited dilution culture was performed with 4T1-luc2-1A4 cells in four 96-well plates. Cells were grown for 10 days and examined their luciferase expression by adding D-luciferin into the culture media. Bioluminescent images were taken immediately. Wells that did not show any luciferase activity did not contain live cells. (5.36 MB TIF) [file pone.0009364.s002.tif]

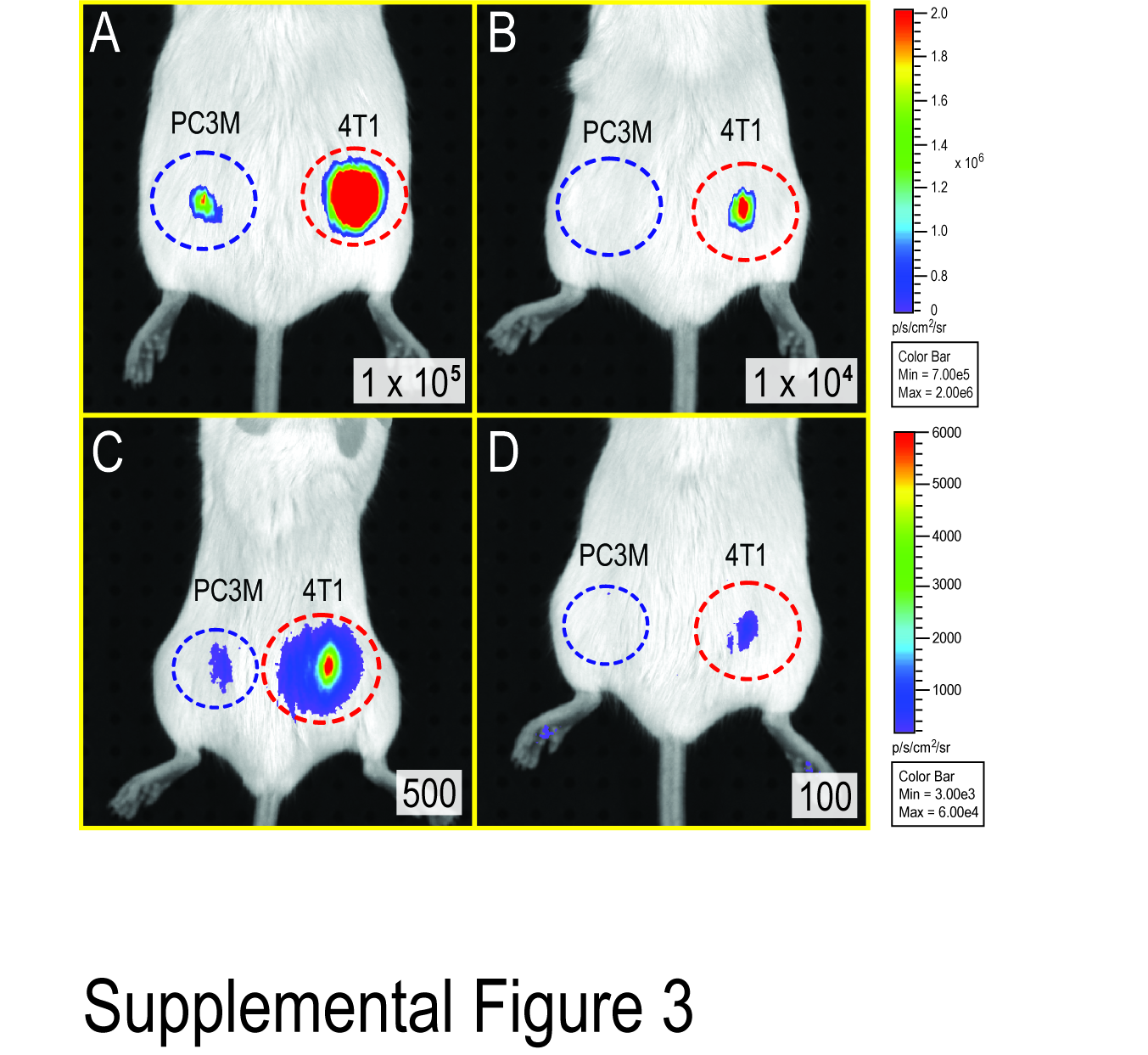

Supplement: Figure S3 — (A-D) The 4T1-luc2-C26 and the PC3M-luc-C6 cells were subcutaneously implanted into flank regions of SCID-bg mice. Equal numbers of cells for each cell line was implanted. Bioluminescence images were taken 20 hrs post-implantation using an IVIS Spectrum. Numbers of implanted cells are shown on the inserts. Imaging conditions (A,B; FOV: B, binning: small, f stop: 1, exposure time: 30 sec; C,D; FOV: B, binning: small, f stop: 1, exposure time: 5 min). (2.72 MB TIF) [file pone.0009364.s003.tif]

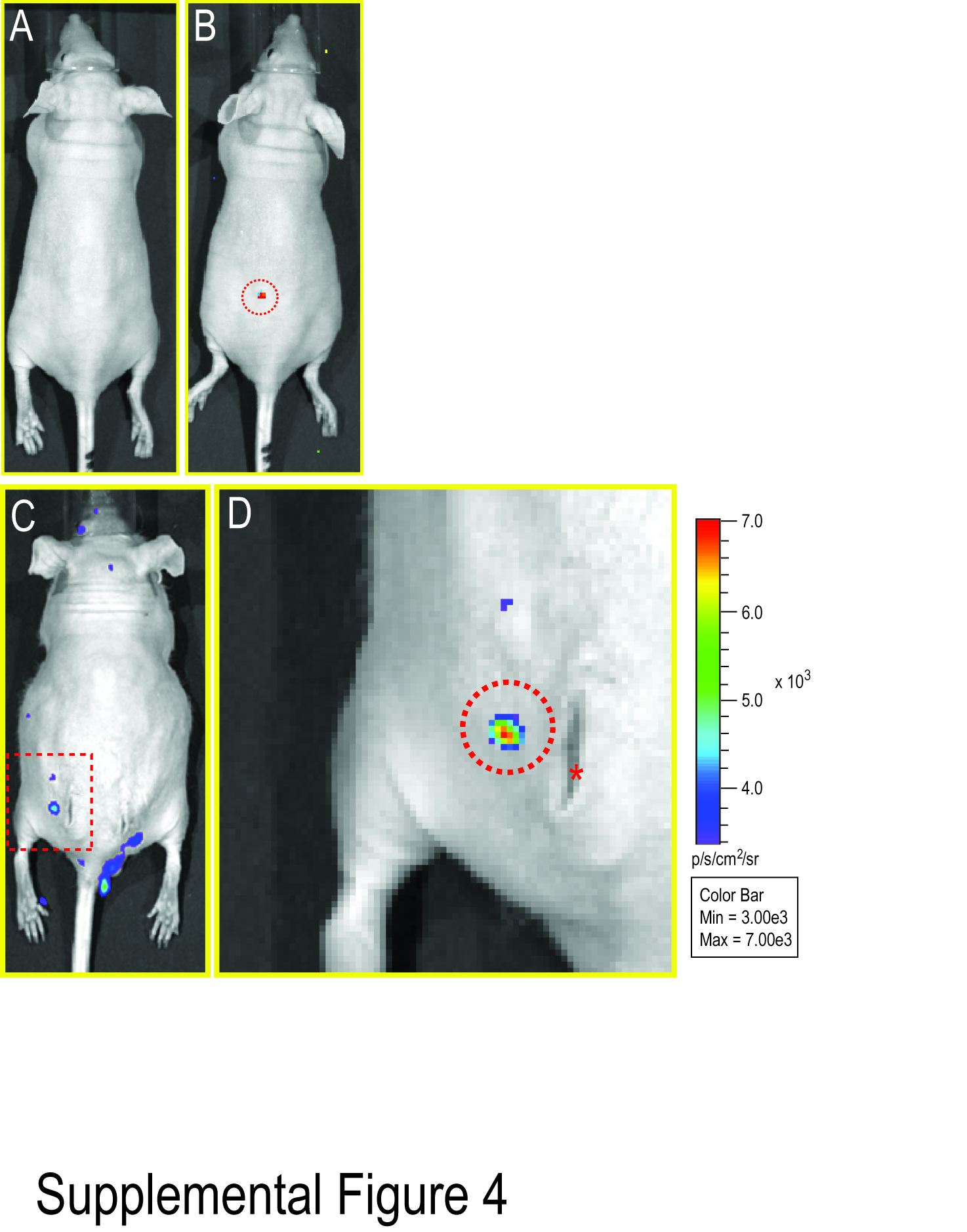

Supplement: Figure S4 — (A, B) Bioluminescent signal of a single 4T1-luc2-1A4 cell in vivo. Female nu/nu mouse was implanted with a single 4T1-luc2 cell subcutaneously in the dorsal region. Mouse was imaged prior to D-luciferin injection (A). Ten minutes after the D-luciferin injection, whole mouse image was taken (FOV; C, binning; small, f stop; 1, exposure time; 5 min) (B). Dotted circle indicates the signal from the implanted cell. (C) Whole mouse (nu/nu) image with implanted ten 4T1-luc2-1A4 cells. The exact number of cells was picked up by a glass capillary pipet and was injected into the back of the mouse subcutaneously, through a skin incision. (D) Magnified image of dotted area from panel C. Dotted circle indicates the signal from 10 cells. Asterisk (*) indicates the skin incision site. (3.57 MB TIF) [file pone.0009364.s004.tif]
